# Imaging Modalities in Medication-Related Osteonecrosis of the Jaw: A Narrative Review of Diagnostic Findings and Staging

**DOI:** 10.3390/medicina61091578

**Published:** 2025-08-31

**Authors:** Marius Ciprian Manole, Mihnea Nicoară, Alexandru Victor Burde, Ioana Hedeșiu, Dan Nicolae Bele, Mihaela Hedeșiu, Florin Crișan, Alexandru Grecu, Cosmin Sinescu, Meda Lavinia Negrutiu

**Affiliations:** 1 Department of Prosthetics and Dental Materials, Faculty of Dentistry, Iuliu Hatieganu University of Medicine and Pharmacy, 400012 Cluj-Napoca, Romania; marius.manole@umfcluj.ro (M.C.M.); grecu.alexandru@umfcluj.ro (A.G.); 2 Doctoral School, Iuliu Hatieganu University of Medicine and Pharmacy, 400012 Cluj-Napoca, Romania; mihnea.ionu.nicoara@elearn.umfcluj.ro (M.N.); florin.crisan@institutulinimii.ro (F.C.); 3 Department of Dental Technology, Faculty of Nursing and Life Sciences, Iuliu Hatieganu University of Medicine and Pharmacy, 400012 Cluj-Napoca, Romania; 4 School of Medicine, University of Liverpool, Liverpool L69 3GE, UK; hlihedes@liverpool.ac.uk; 5 2nd Department of General Surgery, Faculty of Medicine, Iuliu Hatieganu University of Medicine and Pharmacy, 400012 Cluj-Napoca, Romania; nicolae.bele@umfcluj.ro; 6 Department of Oral and Maxillofacial Surgery and Radiology, Faculty of Dentistry, Iuliu Hatieganu University of Medicine and Pharmacy, 400012 Cluj-Napoca, Romania; mhedesiu@umfcluj.ro; 7 Department of Prosthetic Technology and Dental Materials, “Victor Babes” University of Medicine and Pharmacy Timisoara, 300070 Timișoara, Romania; sinescu.cosmin@umft.ro (C.S.); negrutiu.meda@umft.ro (M.L.N.)

**Keywords:** osteonecrosis, bisphosphonates, osteolysis, imaging, cone-beam CT

## Abstract

*Background and Objectives*: Medication-related osteonecrosis of the jaw (MRONJ) is a serious complication of antiresorptive and antiangiogenic therapies. Early and accurate imaging is crucial for diagnosis and management. This review summarizes the current evidence on MRONJ imaging findings across modalities and identifies gaps for future research. *Materials and Methods*: This narrative review analyzed 32 studies (2010–2024) retrieved from PubMed and EBSCO examining imaging findings and diagnostic patterns of medication-related osteonecrosis of the jaw across different modalities. Two independent reviewers screened all articles, extracted data, and assessed methodological quality. *Results*: Early-stage MRONJ findings included osteosclerosis, lamina dura thickening, and increased prominence of the inferior alveolar canal, while late-stage findings included periosteal reactions, sequestration, and cortical erosion. CBCT and MRI were most sensitive for early detection and lesion extent. However, substantial variability in imaging protocols limited direct comparisons between studies. *Conclusions*: This review highlights the variable imaging findings of MRONJ and the need for standardized protocols. Advanced imaging techniques and quantitative indices hold promise for improving early diagnosis, staging, and management.

## 1. Introduction

Antiresorptive agents such as bisphosphonates (BP) and denosumab (DNB) are useful in the treatment of cancer-related conditions [1] and in preventing fractures associated with fragility in individuals with osteoporosis and osteopenia [2,3]. Medication-related osteonecrosis of the jaw (MRONJ) is defined as a clinical condition in which exposed bone in the area of the jaw does not heal within eight weeks in patients who have received antiresorptive treatment alone or in combination with antiangiogenic or immunomodulatory drugs [4].

MRONJ is a severe complication marked by progressive jawbone degradation and structural changes. BPs, particularly pamidronate, are associated with the highest risk (up to 500-fold increase) [5], though other drug classes such as RANKL inhibitors, antiangiogenic agents, and m-TOR inhibitors also contribute [6]. The condition is influenced by comorbidities (e.g., diabetes, anemia, autoimmune diseases) [7], as well as corticosteroid use, chemotherapy [8], and potentially smoking [9]. Cancer patients receiving antiresorptive therapy (BPs or DNB) face a substantially higher risk (up to 8%) [10] compared to osteoporosis patients (<0.05%) [4].

Local factors further modulate MRONJ risk. Tooth extractions are involved in most cases (up to 82%) [11], especially when preceded by periodontal disease [12]. The mandible is more commonly affected than the maxilla (75% vs. 25%) [4], and denture use may exacerbate the risk in oncologic patients [13].

The overall prevalence of MRONJ remains low in patients treated for osteoporosis or nonmalignant bone diseases, with oral BPs (e.g., alendronate) showing a prevalence of 0.02–0.05% and intravenous zoledronate ≤ 0.02% [4,14]. DNB in osteoporosis patients has a slightly higher prevalence, ranging from 0.04% to 0.3% [15], while romosozumab carries a similar risk of 0.03–0.05% [4]. Cancer patients, however, experience a significantly higher prevalence due to frequent use of high-dose antiresorptives and additional contributing factors such as immunosuppression and concurrent antiangiogenic therapies [16]. In this population, intravenous BPs like zoledronate carry a risk of 0–18%, typically clustering below 5%, while DNB has a comparable risk ranging from 0% to 6.9% [4]. Antiangiogenic agents like bevacizumab and tyrosine kinase inhibitors have been linked to MRONJ in isolated cases, but their overall prevalence is much lower [17]. 

The MASCC/ISOO/ASCO guideline categorizes the risk of medication-related osteonecrosis of the jaw based on the intensity of bone-modifying agents and the clinical circumstances [18]. High-intensity BMAs, commonly administered intravenously for bone metastases in cancer patients, are linked to a substantially increased MRONJ risk, particularly when used concurrently with other treatments [4,18]. Conversely, low-intensity BMAs, such as oral bisphosphonates or denosumab used for osteoporosis or as adjuvant therapy, exhibit a considerably lower incidence rate [18].

For individuals receiving high-intensity BMAs, especially those undergoing or commencing cancer therapy, rigorous dental surveillance and proactive preventive measures, including pre-treatment dental assessments, are strongly advised. Patients using low-intensity BMAs, particularly those with additional risk factors or treatment durations exceeding three years, also warrant risk-stratified evaluations and individualized monitoring. Effective management necessitates close collaboration among oncologists, dentists, and oral health professionals throughout the treatment [4,18,19]. In certain regions of the world, the diagnosis and staging of medication-related osteonecrosis of the jaw (MRONJ) continue to rely predominantly on clinical criteria [20]. While medical imaging is commonly used to establish the diagnosis and evaluate the treatment response, there is still no consensus on the utility of various imaging patterns and their correlation with disease staging. However, the optimal treatment plan is not straightforward, as morbidity, disease severity, and extent need to be balanced against surgical risk, and drug holidays are neither an option in some cases nor proven to significantly impact disease progression. As such, it is essential that a system for assessing and, ideally, quantifying disease severity be developed to simplify the decision-making process and allow medical centers to compare their experiences, hopefully as a first step to more robust imaging guidelines. This study aims to summarize the imaging patterns of MRONJ across various radiological modalities and to examine the relationship between imaging findings and clinical presentation by comparing key results and trends reported in the literature.

## 2. Materials and Methods

This narrative review followed the good-practice recommendations for scoping and synthesizing evidence in qualitative (narrative) literature reviews [21]. A comprehensive search of the literature was conducted using PubMed and EBSCO to identify relevant articles published in English between January 2010 and April 2024. Search terms included “bisphosphonate-associated osteonecrosis of the jaw,” “MRONJ,” “BRONJ,” “imaging,” “MRI,” “CBCT,” “cone-beam,” and related terms. The complete search strategy is provided in Appendix A.

Studies eligible for inclusion investigated imaging findings in medication-related osteonecrosis of the jaw among human subjects who had been administered intravenous or oral BPs. The research designs incorporated observational studies, including clinical trials, case–control studies, cohort studies, and cross-sectional analyses. Studies such as case reports, reviews, investigations on animal subjects, laboratory research, and articles focusing on osteonecrosis affecting bones other than the jaw were excluded from the analysis.

The titles and abstracts of all identified articles were screened against predefined eligibility criteria by two independent reviewers. Subsequently, full texts of articles deemed potentially relevant underwent detailed assessment. Disagreements were resolved through discussion, with a third reviewer consulted when necessary. Data extraction was conducted independently by the same two reviewers, utilizing a standardized form to document study design, participant characteristics, interventions, imaging modalities, and quantitative results.

All outcomes of interest were predefined, with a focus on radiologic imaging features of MRONJ, such as cortical disruption, bone sclerosis, sequestration, periosteal reaction, and soft tissue involvement. Data were extracted from all-time points and imaging sequences, if available. In addition, other variables were recorded, such as duration of therapy, drug type (BPs or DNB), underlying disease (e.g., osteoporosis or cancer), MRONJ stage, and imaging protocol details.

Due to substantial heterogeneity in study designs, imaging techniques, and reported outcomes, a meta-analysis was not feasible. As a result, no formal effect measures (e.g., odds ratios or mean differences) were applied. Instead, a qualitative narrative synthesis was conducted. Imaging findings were compared across modalities, MRONJ stages, and patient subgroups to identify diagnostic patterns, clinical implications, and gaps in the literature. The certainty of evidence for each imaging modality and diagnostic feature was evaluated qualitatively, based on study quality, consistency of findings, and clarity of data reporting.

The methodological quality of the included studies was evaluated using a modified version of the Newcastle–Ottawa Scale, assessing three domains: selection of participants (max 4 points), comparability of groups (max 2 points), and quality of outcome/exposure assessment (max 3 points). A summary of these evaluations, along with individual justifications, is presented in Appendix B.

## 3. Results

A total of 32 studies, encompassing 3100 participants, met the predefined inclusion criteria and were incorporated into the final analysis. Among these participants, 1148 were diagnosed with medication-related osteonecrosis of the jaw, while 1952 individuals served as controls. The study designs varied, including retrospective and prospective cohorts, case–control studies, and cross-sectional analyses. The majority of studies focused on patients treated with BPs alone, while several investigations also included individuals receiving DNB or corticosteroids. The duration of antiresorptive therapy varied considerably across studies, ranging from a few months to over ten years. Commonly reported comorbidities among MRONJ cases included osteoporosis, diabetes mellitus, and various malignancies, notably breast cancer, prostate cancer, and multiple myeloma. A detailed summary of the included studies, including reference, publication year, study type, sample size, drug exposure, treatment duration, and major comorbidities, is presented in Table 1.

The reviewed studies show that 18 employed conventional radiography, 21 used cone-beam computed tomography, and 9 explored magnetic resonance imaging. Several investigations incorporated multiple imaging modalities, allowing for comparative assessment. The utility of each modality varied depending on the disease stage. Radiography was commonly used for initial screening but showed limited sensitivity in early MRONJ detection. CBCT was the most frequently employed modality, offering high-resolution, three-dimensional visualization of cortical and trabecular bone changes, particularly useful for assessing the extent of bone involvement in intermediate- and late-stage disease. MRI, although used less frequently, provided valuable insights into early medullary bone changes and soft tissue involvement, potentially making it superior for identifying stage 0 MRONJ or subclinical cases. Table 2 summarizes the distribution of imaging modalities across diagnostic stages, from early identification to advanced disease, as well as their role in treatment planning and follow-up.

The main imaging findings on 2D radiography and CBCT were osteosclerosis (with a prevalence ranging from 24% to 95.6%), osteolysis (23.6% to 95.6%), periosteal reaction (10% to 64%), and sequestrum formation (9.2% to 75%), as can be observed in Table 3. In contrast, the MRI studies revealed great variability in study design, outcomes, and terminology, which, combined with the general paucity of studies, made generalization difficult. Studies that tried to determine lesion detectability showed that CT vastly outperformed OPT [26,40] and even narrowly surpassed MRI [26], whilst MRI was generally better at indicating lesion extent [26,51].

The methodological quality of the included studies was assessed using a modified Newcastle–Ottawa Scale, and the results are summarized in Appendix B. Most studies achieved moderate scores (range: 4–6 out of 9), with only a few reaching high quality (≥7 points), such as Ristow et al. [39] and Torres et al. [41], which included histological validation and matched controls. Common limitations included small sample sizes, absence of control groups (e.g., Demir et al. [27], Garcia-Ferrer et al. [53]), lack of blinding, and minimal adjustment for confounders. Studies employing objective radiographic measurements and structured indices, such as Soundia et al. [34] and Walton et al. [36], generally scored higher in the outcome domain. However, across the dataset, comparability scores were often limited due to inadequate matching or a lack of multivariable analyses. These quality limitations should be considered when interpreting the heterogeneity of imaging findings and diagnostic performance across modalities.

### 3.1. Imaging Patterns in Early Diagnosis of MRONJ (Stage 0)

#### 3.1.1. Panoramic Images and CT/CBCT

Early MRONJ imaging typically reveals osteosclerotic or lytic patterns, along with morphometric alterations visible on plain radiographs and CT scans [23,29,32,34,38,39]. While dental radiographs are widely accessible, their sensitivity is limited, requiring over 40% bone loss for detection [54,55].

Lamina dura thickening, inferior alveolar canal prominence, and osseous sclerosis are common early indicators on panoramic and CT imaging [29]. Trabecular sclerosis (diffuse: 57%; localized: 30%), crater-like defects (70%), and cortical erosion (13%) are also frequent, though no single feature reliably predicts bone exposure [34].

Mixed lytic-sclerotic changes dominate radiographic presentations (68.6%), whereas periosteal reactions are rare (7.1%) [22]. Digital subtraction techniques enhance early detection by revealing sclerotic changes in nearly one-third of cases [38].

#### 3.1.2. MRI Examination

MRI is valuable for detecting early MRONJ changes, particularly cancellous bone involvement and ill-defined lytic areas. Loss of the normal T1 hyperintensity in the mandible and maxilla has been identified as an early marker, preceding bone destruction [56]. Characteristic MRI features include low T1 signal and moderately increased T2/STIR signal, with gadolinium-enhanced sequences revealing mildly enhancing lytic zones and sharply demarcated sequestrum [57].

Quantitative techniques such as diffusion-weighted imaging (DWI) and apparent diffusion coefficient (ADC) mapping have shown promise. With an ADC cutoff of 1.06 × 10^3^ mm^2^/s, stage 0 MRONJ could be differentiated from controls with high sensitivity (0.89), specificity (0.9), and AUC values up to 0.94, supporting its utility for early tissue characterization [51].

Dynamic contrast-enhanced MRI (DCE) has demonstrated perfusion abnormalities in MRONJ, correlating with qualitative findings from UTE-MRI. Significant parameters included wash-in rate, peak perfusion, and time to peak, though no differences were linked to lesion morphology (osteolytic vs. sclerotic) [52].

Both CT and MRI outperform panoramic radiographs in early lesion detection (accuracy: 96% and 92% vs. 54%), though both tend to overestimate lesion extent intraoperatively [25]. Similarly, while CBCT, MR+CEMR, and PET/CT improve detection over clinical evaluation alone, only PET/CT significantly overestimates lesion size compared to surgical findings [50].

### 3.2. Imaging Findings for Late Diagnosis of MRONJ (Stages I–III)

#### 3.2.1. Panoramic Images and CT/CBCT

In advanced MRONJ (stages I–III), imaging typically reveals sclerosis, osteolysis, sequestration, and periosteal reaction, with the latter more frequently associated with stage III disease [36]. Panoramic radiographs can identify lamina dura thickening and inferior alveolar canal prominence but are inferior to CT in detecting cortical perforation and periosteal deposition [29].

CBCT has shown significantly higher diagnostic accuracy than panoramic radiography for identifying non-vital bone (AUC: 0.88 vs. 0.562, *p* < 0.001) [39], though both modalities visualize stage III-related fractures similarly. Fractal dimension analysis failed to differentiate patients with or without bone exposure despite clear radiographic findings like osteolysis and sclerosis [33].

Quantitative morphometric analysis improves CT-based staging by measuring changes in cortical and trabecular structures. Indicators such as elevated mandibular cortical index (MCI) [23], reduced mandibular canal diameter [28], and decreased bone density and width near the mental foramen [44] support MRONJ diagnosis, although some features (e.g., incisive canal changes) lack discriminatory power.

Measurements of cortical thickness around the mental foramen demonstrated high reproducibility and strong association with MRONJ presence [41]. CT-based classification systems using MCI grades and trabecular sclerosis have also shown potential to predict disease progression, particularly in distinguishing BRONJ from BP-only effects [31]. Cortical thickness ratios further differentiated healthy, BP-treated, and MRONJ patients [42].

Panoramic radiography detects MRONJ lesions in only approximately 54% of cases, while CT and MRI yield much higher accuracy (96% and 92%, respectively), independent of clinical stage [25,27]. MRI remains superior for evaluating soft-tissue extension, bone marrow involvement (92.9%), and sinus lesions (21.4%).

#### 3.2.2. MRI Examination

MRI complemented CT in MRONJ stage III by highlighting adjacent soft tissue involvement, seen as areas of high STIR signal intensity. Bedogni et al. [58] found that MRI, in conjunction with CT, correlated well with histopathology in describing the characteristic lesions of MRONJ. Low water content, seen as universally low T1, T2, and STIR signal, was associated with necrosis and was dominant in the exposed bone, while the high-water content pattern (low T1; high T2 and STIR) indicated bone edema and was present in unexposed but affected jaw tissue. Advanced stages of the disease exhibited both patterns, the second one being peripherally distributed in relation to the first. 

Krishnan et al. [56] described several soft tissue signal abnormalities, including soft tissue edema and enhancement, inferior alveolar nerve thickening, and pterygoid muscle swelling and enhancement. This can be augmented using ultrashort echo time (UTE-MRI) sequences such as the protocol put forward by Huber et al. [49]. These novel sequences allow the visualization of structures such as ligaments, joints, and bones and were shown to be equivalent to CBCT in describing bone lesions when artifacts were accounted for.

### 3.3. Imaging Pattern of MRONJ in the Presence of Comorbidities, Other Medications, and Dental Infections 

Several imaging features appear more frequently in patients treated with antiresorptive agents, including sclerosis, visible alveolar sockets, enhanced lamina dura and mandibular canal, periosteal reaction, and cortical osteolysis, particularly in oncologic patients compared to those treated for non-malignant conditions [30].

Widening of the periodontal ligament (PDL) and generalized bone loss have also been investigated as potential radiographic biomarkers. In patients treated with zoledronic acid, PDL widening was noted in 25% of MRONJ cases (with 75% generalized), while multiple myeloma patients more often showed localized changes (96%). The average periodontal bone loss reached 30.2% [37].

Comparisons between cancer patients treated with zoledronate and healthy controls revealed radiographic changes in the alveolar cortex, medullary bone, and dentoalveolar region, most commonly in the posterior mandible (45.3%), likely due to local vascular differences. However, associations with cancer type or regimen remained inconclusive [32].

The route of drug administration appears to influence radiographic patterns, as buccolingual cortical perforations were more frequently seen in patients receiving intravenous BPs, although no clear correlation was found with treatment duration or clinical staging [40].

CBCT evaluations following tooth extraction have identified imaging features strongly associated with subsequent MRONJ development. Findings such as lamina dura thickening, widened PDL space, osteosclerosis, osteolysis, and sequestration were notably present in patients who later developed osteonecrosis. Substantial inter-reader agreement (κ = 0.69) confirmed the reliability of this diagnostic framework [43].

### 3.4. Imaging Pattern of MRONJ in the Presence of Osteomyelitis (OM) and Osteoradionecrosis (ORN)

Given the similarities between the histopathologies of MROJN, OM, and ORN, a certain degree of radiological feature overlap is to be expected. Both conventional 2D radiography and CBCT tend to reveal the same patterns of structural bone changes with varying degrees of diagnostic accuracy [42]. Imaging examinations can distinguish between active osteonecrosis and inflammation [59,60]. The mandibular cortical index (MCI) was significantly higher in MRONJ than in OM [25]. Sequestrum formation together with extraction sockets was more commonly encountered in MRONJ, whereas periosteal new bone was a better marker of OM [24].

In MRONJ, CBCT scans commonly reveal internal bone textures that are both lytic and sclerotic, along with sequestration, buccal or lingual periosteal reactions, and cortical perforation. These characteristics are observed more frequently than in ORN, where periosteal changes are typically absent [45].

To standardize assessment, composite radiographic indices (CRIs) have been proposed. One such CRI scores sclerosis, lysis, periosteal reaction, and sequestration (0–2 per feature), yielding a total between 0–8. Scores above 6 correlate with advanced clinical staging [36]. Another version, incorporating trabecular sclerosis, cortical erosion, periosteal changes, and crater-like defects, showed that sequestration was predictive of progression: present in 90% of cases that advanced to bone exposure, but only 20% in limited stage 0 disease [34].

Yfanti et al. [46] proposed an expanded CRI that incorporated eight features, including sinus involvement, non-healing sockets, and jaw fractures, to differentiate MRONJ and OM from jaw metastases. However, the generalizability of this index is limited due to its dependence on handcrafted imaging features.

Advanced approaches using radiomics and texture analysis have also emerged. A CT-based model incorporating gray-level run length and zone length matrix features achieved promising diagnostic performance for staging MRONJ by capturing bone marrow changes [47].

## 4. Discussion

This review focused on structural imaging findings of MRONJ as evidenced through clinical studies. The modalities that were discussed were dental radiographs, CT/CBCT, and MRI, with the first two being part of routine practice in dentistry and the latter enjoying widespread use in general medicine and gradually being adapted to the needs of the dental community. The studies included in our analysis reflect this fact: only 29 out of the 32 studies described CT or 2D radiological findings, and only 3 studies focused solely on MRI. Studies that compared CT with MRI (five studies) highlight the ongoing need to validate the more expensive and time-consuming examination as a practical alternative to the established standard. 

Different imaging modalities offer distinct advantages and trade-offs. Dental radiographs and CBCT remain the first-line diagnostic tools due to their accessibility and cost-effectiveness. However, their limitations, such as the need for significant bone loss to detect changes, restrict their utility in early-stage diagnosis. Studies like those by Stockmann et al. [25] and Ristow et al. [39] have demonstrated the superior accuracy of CT and MRI in detecting lesions compared to panoramic radiography.

MRI, though underutilized, offers unique advantages in soft tissue characterization and early bone marrow changes. For example, Krishnan et al. [56] and Chiandussi et al. [57] reported that loss of the normal T1 hyperintensity signal and the presence of ill-defined lytic areas on T2/STIR sequences are early signs of MRONJ. Additionally, the use of advanced techniques such as diffusion-weighted imaging (DWI) and apparent diffusion coefficient (ADC) analysis, as demonstrated by Muraoka et al. [51], has shown potential in distinguishing MRONJ from normal bone with high sensitivity and specificity.

Imaging findings such as osteosclerosis, osteolysis, periosteal reaction, and sequestration provide critical insights into the disease’s pathophysiology and progression. For example, Walton et al. [36] highlighted that these features vary across MRONJ stages, with periosteal reaction more commonly associated with stage III disease. Moreover, morphometric measures like the mandibular cortical index (MCI) and trabecular bone sclerosis have been proposed as quantitative biomarkers for MRONJ, as explored by Torres et al. [41] and Kubo et al. [31].

Imaging modalities are indispensable for monitoring treatment responses and disease progression. Features like sequestration and cortical irregularity, as reported by Ogura et al. [45], are indicative of advanced disease and may guide surgical planning. Walton et al. [36] proposed the Composite Radiographic Index (CRI) as a tool to quantify disease severity based on imaging findings, correlating higher scores with advanced clinical stages.

To support clinical decision-making, we synthesized data from the included studies into a comparative summary of each imaging modality’s diagnostic limitations, accessibility, and institutional impact. Table 4 provides an overview of key features relevant to MRONJ evaluation in daily practice, helping contextualize imaging choices within real-world settings.

Additionally, imaging plays a role in evaluating the impact of treatment interventions, such as drug holidays or surgical procedures. For instance, Soundia et al. [34] found that sequestrum was predictive of progression to exposed bone disease, emphasizing the importance of imaging in treatment planning and prognostication.

### 4.1. Challenges and Future Directions

The variability in imaging findings across studies highlights the need for standardized protocols and scoring systems. The lack of consensus on imaging patterns and their correlation with clinical stages impedes the development of universal diagnostic and management guidelines. Future research should focus on establishing such standards, particularly for advanced imaging techniques like MRI and PET/CT.

Functional imaging (e.g., SPECT and PET) is a promising avenue for MRONJ diagnosis, as it can reveal metabolic changes beyond the detection capabilities of structural imaging. Single-photon emission computer tomography can create a 3D metabolic map of the entire body, with every voxel having a specific uptake value (SUV), using molecules known as tracers to target specific tissues. Spatial resolution can be further enhanced in conjunction with CT imaging (SPECT/CT). Some of the available radiotracers helpful in imaging MRONJ are 99Tcm-DPD, 99Tcm-MDP, and 99mTc-HMDP. In a recent small study, 18F-fluoride PET showed markedly higher uptake in MRONJ-affected bone than 18F-FDG, corresponding with greater osteocyte density on histology, whereas micro-CT revealed no significant microarchitectural differences [60]. Nuclear imaging can reflect pathophysiological changes such as necrosis, inflammation, and vascular remodeling [66], but SPECT/CT’s limited spatial resolution may hinder precise tissue correlation [67].

Furthermore, functional imaging findings can have clinical significance. The sensitivity of SPECT/CT has outpaced CT and MRI imaging for quite some time [63], and, at present, there is even software that can successfully predict the diagnosis of stage II MRONJ up to three months in advance [45], proving that this technique can be integrated into the paradigm of quantitative imaging. Maximum SUV has emerged as a method to numerically express uptake and has yielded promising results both in diagnosis [68] and in surgical planning [69]. In a paper published in 2021, Ogawa and Ogura [69] showed that the mean maximum SUV was significantly higher in MRONJ patients with osteoporosis compared to cancer patients. 

Of course, there is still much room for improvement. PET/CT has started to be investigated as a potential way to combine the high sensitivity of functional imaging with the fidelity and spatial resolution of conventional imaging techniques. Abnormal FDG uptake was demonstrated in MRONJ-affected bone when compared with normal jaw [65], and SUVmax, as measured on PET/CT, can indicate refractory disease [70].

When comparing the clinical applications of functional and structural imaging, Guggenberger et al. [50] concluded that, out of the three imaging modalities compared (panoramic views from CBCT, MR+CEMR, and PET/CT), only PET/CT significantly overestimated disease extent as measured during intraoperative assessment, and all three were better than preoperative assessment alone.

Radiomics and artificial intelligence (AI) offer promising avenues for addressing these challenges. Textural analysis and machine learning models, such as that by Ito et al. [47], focused on the CBCT appearance of affected versus healthy bone. However, these technologies require further validation and standardization before widespread clinical adoption. A 2023 systematic review of 23 papers by Santos et al. [70] noted that most focused solely on disease diagnosis or classification, with limited standardization and no studies addressing response prediction, prognosis, or therapeutic outcomes.

As of the writing of this article, no validated imaging-based deep learning model for the prediction of MRONJ exists. However, this approach could lead to promising results, as was the case for osteonecrosis of the hip, where such models showed comparable diagnostic accuracy to experienced orthopedic surgeons [71]. Work on PET/CT aiming to provide a more quantitative measure of bone lesions has employed the bone scan index (BSI), a measure of uptake relative to the entire skeleton, and could prove fruitful in predicting the development of MRONJ [65].

Similarly, micro-CT studies, such as those by Schoenhof et al. [72], have provided valuable insights into the microarchitectural changes associated with MRONJ, shedding light on the disease’s early physiopathology. These findings could inform the development of imaging biomarkers and guide therapeutic interventions. In their study, Schoenhof et al. performed a morphometric analysis of 141 bone samples extracted from 78 patients using micro-CT and concluded that, while antiresorptive medication could decrease bone density loss through inhibition of osteoclastic activity, it did little to prevent loss of bone tissue connectivity. The ensuing accumulation of microcracks caused by repeated mechanical stress at the level of the jawbones was thus the main driver behind the onset and progression of the disease in the early phase.

### 4.2. Limitations

The present review highlights several limitations that restrict the generalizability and clinical applicability of the findings. Patient cohorts varied in clinical indication, antiresorptive drug type, treatment duration (from a few months to over ten years), and MRONJ stage distribution. Imaging protocols were inconsistent, encompassing diverse modalities with variable technical parameters (e.g., CT voxel resolution, MRI sequence type) and heterogeneous image analysis or scoring methods. Definitions of diagnostic outcomes also varied: studies assessed different radiographic parameters of disease extent or severity and often applied non-uniform MRONJ staging or lesion classification systems. All 32 studies were observational and non-randomized (Levels 3–4 evidence per Oxford criteria [73]; low to very low certainty by GRADE [74]), with most being small, retrospective case series or cohort designs. Common limitations included lack of blinding, absence of comparator groups, and failure to control for confounding variables. Only a minority performed multivariable analyses (e.g., Baba et al. [40]; Kubo et al. [31]; Rocha et al. [32]) or used histologic confirmation of imaging findings (e.g., Ristow et al. [39]; Stockmann et al. [25]). NOS scores ranged from 2 to 7/9, with most studies scoring 2–5, reflecting limited internal validity.

Imaging protocols and outcome definitions varied considerably, complicating cross-study comparisons. Inconsistencies were noted in voxel size, MRI sequences, image scoring, and MRONJ staging systems. Emerging modalities (e.g., texture analysis [47], UTE-MRI [49], [^18F]-PET/CT [50]) were exploratory, with small samples and no validation cohorts. It is, however, important to stress that several prior reviews and expert position papers concur with the present assessment that the imaging literature on medication-related osteonecrosis of the jaw (MRONJ) is characterized by low-quality evidence and substantial methodological heterogeneity. Foundational imaging studies by Krishnan et al. [56] and Chiandussi et al. [57] introduced early radiologic and MRI patterns of MRONJ but were limited to small case series without controls. Consistent with our findings, subsequent systematic reviews and expert consensus statements have noted that most MRONJ imaging studies remain retrospective, single-center designs lacking randomization, blinding, or standardized imaging protocols [7,18,55]. The 2019 MASCC/ISOO/ASCO Clinical Practice Guidelines acknowledged the insufficiency of imaging data for establishing reliable diagnostic strategies, instead relying on expert consensus to guide practice [18]. The 2022 update of the AAOMS Position Paper reiterated that MRONJ exhibits a broad spectrum of radiologic presentations and cautioned against over-reliance on imaging alone, particularly in early-stage disease [55]. Furthermore, Khan et al. [54], in an international consensus document, highlighted both the utility and limitations of radiographic modalities while emphasizing that no imaging findings are pathognomonic. Small sample sizes in several studies further limit the statistical power of their findings, emphasizing the need for large-scale, prospective research to validate and extend the insights gained from this review.

## 5. Conclusions

Imaging is essential for diagnosing and managing MRONJ, but current approaches vary across different types. Differences in imaging results, terminology, and diagnostic thresholds mirror the different protocols, study designs, and disease presentations. Standard tools like panoramic radiography and CBCT are common, but they are limited in identifying the disease in its early stages. Newer methods, such as PET/CT and radiomics, could offer quantifiable indicators of the disease, but they are not standardized or routinely employed yet.

To enhance diagnostic accuracy and clinical decision-making, there is a clear need for unified imaging criteria, validated scoring systems, and greater integration of functional and structural imaging. Future large-scale, prospective studies are essential to establish the reliability and clinical utility of these advanced techniques, ultimately contributing to earlier detection, more accurate staging, and improved outcomes for MRONJ patients.

## Figures and Tables

**Table 1:** Summary of study methodologies and patient characteristics.

Reference	Year	Type of the Study	No. of Patients (Cases/Controls)	Drugs	Length of Therapy	Main Comorbidities
Shin et al. [22]	2019	Retro. cohort	161/179	BP + DNB	4.5 y avg.	HTN, OP, DM
Hutchinson et al. [23]	2010	Case–control	30/975	BP + DNB	Median 3.0 y (2.4–4.7)	OP, PCa, MM
Kammerer et al. [24]	2016	Cross-sectional	14	BP	Not specified	OP, SLE, RA, DM
Stockmann et al. [25]	2009	Cross-sectional	28	BP	Not specified	MM, other malignancies, OP
Assaf et al. [26]	2018	Cross-sectional	32	BP + DNB, (+CS in 25%)	Not specified	Multiple systemic diseases (hyperthyroidism, arrhythmias, cancers, etc.)
Demir et al. [27]	2017	Cross-sectional	27	BP	1 to 10 y	DM
Goller-Bulut et al. [28]	2018	Case–control	56/56	BP	Not specified	MM, PCa + BC w/metastases
Guo et al. [29]	2016	Cross-sectional	40	BP	Mean: 37.92 (10–96 mo).	BC w/mets, MM, OP
Klingelhoffer et al. [30]	2016	Case–control	60/60	BP	~4.75 y	OP, bone mets, MM
Kubo et al. [31]	2017	Case–control	24/179/200 B–	BP	67.9 ± 37.1 mo (ONJ +) vs. 40.4 ± 47.9 (ONJ–)	OP, bone mets
Rocha et al. [32]	2012	Case–control	30/30	BP	16 mo	Met. cancers, periodontal dz, history of oral surgery
Sahin et al. [33]	2019	Cross-sectional	66	BP + DNB	21 mo (early); 48 mo (adv)	OP, cancers (BC, lung, PCa, etc.), MM
Soundia et al. [34]	2018	Cross-sectional	23	BP + DNB	52 mo	OP, cancers, sarcoma, GCT
Treister et al. [35]	2009	Cross-sectional	39 (234 sextants)	BP	Not specified	Multiple cancers, Gaucher’s dz, OP
Walton et al. [36]	2019	Cross-sectional	70	BP + DNB	Not specified	Multiple cancers, OP
Wazzan et al. [37]	2018	Case–control	16/100	BP	2 y vs. 1 y	MM, smoking, DM, steroid use
Zaman et al. [38]	2013	Cohort	43	BP	17–74 mo	BC, hepatic cancer, MM, OP
Ristow et al. [39]	2021	Retro. cohort	130	BP + DNB	47.9 mo (1–189)	Multiple cancers, OP
Baba et al. [40]	2018	Prosp. cohort	74	BP + DNB	0.2–15 y (avg. 3.44 y)	Multiple cancers, OP
Torres et al. [41]	2012	Case–control	12/64	BP	Not specified	Cancer-related bone metastases
Koo et al. [42]	2018	Case–control	63/32	BP + DNB	6.3 ± 5.3 y vs. 4.8 ± 4.4 y	Malignant and benign bone conditions
Moreno-Rabie et al. [43]	2023	Case–control	47/50	BP + DNB	MRONJ +: 40.4 mo; MRONJ–: 29.5 mo	BC, MM, PCa, renal cancer, other cancers
Gönen et al. [44]	2018	Case–control	25/25	BP	1–120 mo	Not reported
Ogura et al. [45]	2021	Cross-sectional	10	BP + DNB	Not specified	Met. cancers
Yfanti et al. [46]	2023	Retro. cohort	335	BP + DNB	Not specified	OP, bone mets, oral surgeries
Ito et al. [47]	2021	Case–control	25	BP	4–9 y (avg. ~6.5 y)	Steroids, DM
Sakamoto et al. [48]	2023	Retro. cohort	18	BP + DNB	9–24 mo (non-osteolytic); 25.8 ± 19.3 mo (osteolytic)	DM, steroids, cancers, OP
Huber et al. [49]	2019	Cross-sectional	19	BP	Not specified	Cancers, DM
Guggenberger et al. [50]	2013	Cross-sectional	10	BP	Median 48 mo (7–60 mo)	Cancers, OP
Muraoka et al. [51]	2021	Case–control	38/10	BP + DNB	Not specified	OP/osteopenia, bone mets
Schumann et al. [52]	2022	Cross-sectional	20	BP + DNB	Not specified	OP, PCa, other cancers
Garcia-Ferrer et al. [53]	2008	Cross-sectional	14	BP	18–60 mo (~32.4 ± 14.7)	OP, cancers

Abbreviations: BP—bisphosphonates; DNB—denosumab; OP—osteoporosis; BC—breast cancer; PCa—prostate cancer; MM—multiple myeloma; DM—diabetes mellitus; RA—rheumatoid arthritis; SLE—systemic lupus erythematosus; CS—corticosteroids; mets—metastases; ONJ—osteonecrosis of the jaw; dz—disease; y—years; mo—months.

**Table 2:** Imaging techniques used in the evaluation of MRONJ.

Modality	Early Diagnosis (Stage 0)	Late Diagnosis (Stages I–III)	Management and Follow-Up
X-Ray	[24,25,28,29,33,35,39,40]	[23,27,28,29,30,31,32,34,36,37]	[24,25,26]
CBCT	[24,35,40,44,48]	[28,29,30,32,37,41,42,43,44,45,46,47,50,51]	[24,25,26]
MRI	[48,52]	[49,51,52,53,54]	[26]

Notes: Ranges represent consecutive citations as listed in the References section.

**Table 3:** Absolute frequency of major imaging patterns in early- and late-stage MRONJ.

	Early	Late
Osteosclerosis	120	348
Osteolysis	58	360
Periosteal reaction	19	115
Sequestration	29	296
Lamina dura thickening	31	143
Cortical erosion/irregularity	29	7
Persistent socket	19	59
Prominent inferior alveolar canal	7	95
Fragmentation/fracture	0	14

**Table 4:** Overview of diagnostic performance and feasibility of imaging methods in MRONJ.

Imaging Method	Diagnostic Limitations	Accessibility and Daily Use	Economic and Organizational Considerations
Panoramic radiography	Low sensitivity (~54%) for early MRONJ [1,2]. Requires major bone loss to detect changes, missing early marrow involvement. Two-dimensional images fail to show cortical breaches or sequestra [3,55].	Common, low-cost tool in dental practice with minimal radiation [5]. Often insufficient alone; advanced imaging needed for full evaluation [4].	Low-cost, in-office imaging; no referral needed. Common first-line tool with minimal logistical demands. May necessitate follow-up with advanced imaging, adding downstream costs.
Conventional CT	Detects bone lesions well (~96%) [1], but lacks soft-tissue/marrow detail [6]. May overestimate necrosis; artifacts from dental metals reduce accuracy [7,8].	Available in hospitals, costly, and high-radiation. Used mainly in advanced cases or surgical planning, not routine screening [25,40,50].	CT entails higher institutional costs and radiation exposure compared to dental imaging [61]. It requires referral to hospital-based radiology, often causing delays and additional coordination. Despite its diagnostic benefits, logistical and economic constraints limit its routine use.
Cone-Beam CT	High-resolution 3D bone imaging, more accurate than panoramic X-rays (~88% vs. ~56% AUC) [9]. Cannot show marrow edema; artifacts possible [24,27,33,39].	Widely available in dental settings, lower cost/dose than CT. Preferred for jaw evaluation but limited for extensive or soft-tissue disease.	CBCT is more cost-effective and accessible than conventional CT, and often available within dental or OMFS practices [61]. It minimizes referral burden and integrates efficiently into outpatient workflows, supporting faster diagnosis with lower organizational complexity.
Magnetic Resonance Imaging (MRI)	Best for early marrow and soft-tissue changes in MRONJ [6]. Poor for fine bone detail; variable image quality and dental artifacts limit use [8].	Not available in dental offices. Time-consuming and costly, used selectively for early or soft-tissue cases with inconclusive CT [25,50,56].	MRI demands specialized facilities and higher costs, both for acquisition and scheduling. Access is restricted to imaging centers or hospitals, posing significant logistical barriers. Its use is typically limited to complex cases due to these institutional challenges [62].
Bone Scintigraphy/SPECT	High sensitivity to early changes [12] but low specificity [13,14]. Limited spatial detail; false positives are common. SPECT improves localization [14].	Requires nuclear medicine setup and IV tracer. Time-intensive and moderately radioactive; reserved for complex or unclear cases [15,45,63,64].	SPECT requires nuclear medicine infrastructure and radiotracer logistics, which limits access to tertiary centers. Multistep procedures increase organizational burden, reducing feasibility for routine assessment despite high sensitivity [62].
PET/CT	Shows metabolic activity in MRONJ [16], but uptake overlaps with infections or healing. Tends to overestimate lesion extent; lacks standard criteria [13].	Expensive, high-radiation, limited to tertiary centers. Rarely used in routine care—mostly for oncology or unresolved cases [50,63,65].	PET/CT involves substantial institutional investment in tracers, scanners, and staff. Its complexity restricts availability to major hospitals or research centers. Used selectively; its cost and workflow demands preclude routine clinical application in MRONJ [50].

## Data Availability

No datasets were generated or analyzed during the current study.

## Abbreviations

The following abbreviations are used in this manuscript:

ADC	apparent diffusion coefficient
AI	artificial intelligence
AUC	area under the curve
BC	breast cancer
BMD	bone mineral density
BP	bisphosphonates
BRONJ	bisphosphonate-related osteonecrosis of the jaw
CBCT	cone-beam computed tomography
CRI	composite radiographic index
CS	corticosteroids
CT	computed tomography
DNB	denosumab
DM	diabetes mellitus
DWI	diffusion-weighted imaging
JM	jaw metastases
m-TOR	mammalian target of rapamycin
MDP	technetium-99m-methylene diphosphonate
MM	multiple myeloma
MRI	magnetic resonance imaging
MRONJ	medication-related osteonecrosis of the jaw
OM	osteomyelitis
OPT	orthopantomogram (panoramic radiograph)
ONJ	osteonecrosis of the jaw
ORN	osteoradionecrosis
PCa	prostate cancer
PET/CT	positron emission tomography/computed tomography
PET/MRI	positron emission tomography/magnetic resonance imaging
RA	rheumatoid arthritis
RANKL	receptor activator of nuclear factor kappa-B ligand
ROC	receiver operating characteristic
SLE	systemic lupus erythematosus
STIR	short tau inversion recovery
SUV	standardized uptake value
SPECT	single-photon emission computed tomography
T1	T1-weighted MRI signal
T2	T2-weighted MRI signal
UTE-MRI	ultrashort echo time magnetic resonance imaging
99mTc-DPD	technetium-99m-dicarboxypropane diphosphonate
99mTc-HMDP	technetium-99m-hydroxymethylene diphosphonate
3D	three-dimensional

## Appendix A

**Table A1:** Detailed search strategy for systematic review.

Variable	Search Strategy
Database searched	MEDLINE (accessed through PubMed) and EBSCO databases from their origin until 23 January 2024
Search strategy for PubMed	(“Bisphosphonate-Associated Osteonecrosis of the Jaw/diagnostic imaging”[Mesh]) OR ((“osteonecrosis “OR”MRONJ“OR”BRONJ”) AND “jaw” AND (“medication” OR “drug” OR “bisphosphonate” OR “alendronate” OR “ibandronate” OR “risedronate” OR “zoledronate” OR “zoledronic acid” OR “antiresorptive” OR “Diphosphonates”[MeSH]) AND (“Diagnostic Imaging”[Mesh] OR “Imaging” OR “MRI” OR “magnetic resonance” OR “CBCT” OR “cone-beam” OR “OPT” OR “radiography” OR “X-ray”))
Search strategy for EBSCO	((((“medication-related osteonecrosis of the jaw” OR MRONJ OR BRONJ) AND (imaging OR radiography OR MRI OR CBCT OR “cone-beam computed tomography”))))
Other sources	The reference lists of selected articles and reviews were hand-searched to identify any additional relevant articles.

## Appendix B

**Table A2:** Quality assessment of included studies based on a modified Newcastle–Ottawa Scale (NOS).

Study	Selection *	Comparability *	Outcome/Exposure *	Total *	Justification/Notes
Assaf et al. [26]	2	1	2	5	Matched controls; objective measurement of periodontal ligament width; no control for systemic confounders.
Baba et al. [40]	3	1	2	6	Multivariate logistic regression performed; blinded CT assessment; no control group included.
Demir et al. [27]	1	0	1	2	Case series with minimal sample; no control group; unblinded imaging assessment.
Garcia-Ferrer et al. [53]	1	0	1	2	Descriptive MRI case series; no comparator or validation of outcome.
Goller-Bulut et al. [28]	2	1	2	5	Matched case–control; blinded measurement of canal diameters; no multivariate adjustment.
Gonen et al. [44]	2	1	2	5	Small matched cohort; objective cortical measurement; unclear blinding.
Guggenberger et al. [50]	2	1	2	5	Prospective design; blinded imaging assessment; no control group.
Guo et al. [29]	1	0	1	2	Retrospective comparison; no control group; descriptive imaging analysis.
Huber et al. [49]	2	1	2	5	CBCT vs. UTE-MRI; blinded reviewers; absence of control group and confounder adjustment.
Hutchinson et al. [23]	2	1	1	4	The study highlights radiographic features of stage 0 MRONJ but lacks a control group and uses a small, self-selected cohort
Ito et al. [47]	2	0	2	4	Texture analysis of stage 0 MRONJ; no external control group; objective but unvalidated features.
Kammerer et al. [24]	2	0	1	3	Retrospective CBCT/OPT study; small sample; unblinded evaluation.
Klingelhoffer et al. [30]	2	1	2	5	Matched cases; randomization and blinding of radiographs; limited confounder control.
Koo et al. [42]	2	1	2	5	Cross-sectional comparison; clear inclusion; group comparisons only.
Kubo et al. [31]	3	2	2	7	Matched controls; analyzed morphometric variables; limited statistical adjustment.
Moreno-Rabie et al. [43]	2	1	2	5	Case–control with 3 groups; substantial inter-observer agreement; no multivariable analysis.
Muraoka et al. [51]	2	1	2	5	ADC-based MRI study; control group included; no blinding or full confounder adjustment.
Ogura et al. [45]	2	1	2	5	Surgical specimens compared to ORN; histopathologic gold standard; no multivariable control.
Ristow et al. [39]	3	2	3	8	Histology-validated diagnostic accuracy study; blinded examiners; no adjustment for stage.
Rocha et al. [32]	2	1	2	5	Oncology vs. control patients; radiographic signs compared; no multivariable control.
Sahin et al. [33]	2	1	2	5	Stratified MRONJ severity; no control group; small sample.
Sakamoto et al. [48]	1	0	2	3	Compared osteolytic patterns; no confounder adjustment or control group.
Schumann et al. [52]	2	1	2	5	UTE-MRI perfusion correlation; no adjustment for confounders.
Shin et al. [22]	3	2	2	7	Large cohort with antiresorptive exposure; stratified analyses by imaging features; no follow-up.
Soundia et al. [34]	2	1	2	5	Blinded imaging index for stage 0 progression; small cohort; no adjustment.
Stockmann et al. [25]	2	1	2	5	Compared CT/MRI to intra-op findings; no confounder control; prospective.
Torres et al. [41]	3	2	3	8	CBCT-based morphometry; matched controls; strong reliability metrics.
Treister et al. [35]	2	0	2	4	Descriptive analysis of BONJ imaging signs; no controls or blinding.
Walton et al. [36]	2	1	2	5	Composite index scoring; no multivariable analysis.
Wazzan et al. [37]	2	1	1	4	Retrospective controls; matching methods unclear; poor methodological detail.
Yfanti et al. [46]	2	1	2	5	CRI-based severity score; no external validation; limited confounder adjustment.
Zaman et al. [38]	2	1	1	4	Small radiograph comparison study; unblinded, limited reproducibility.

* Scoring criteria: Selection (max. 4 points), Comparability (max. 2 points), and Outcome/Exposure Assessment (max. 3 points), for a total maximum score of 9. Individual justifications are provided to ensure transparency in scoring.
